# Triggers of intensive care patients with palliative care needs from nurses’ perspective: a mixed methods study

**DOI:** 10.1186/s13054-024-04969-1

**Published:** 2024-05-28

**Authors:** Manuela Schallenburger, Jacqueline Schwartz, Andrea Icks, Jürgen in der Schmitten, Yann-Nicolas Batzler, Stefan Meier, Miguel Mendez-Delgado, Theresa Tenge, Martin Neukirchen

**Affiliations:** 1 Interdisciplinary Centre for Palliative Medicine, Medical Faculty, University Hospital Düsseldorf, Heinrich-Heine-University Düsseldorf, Düsseldorf, Germany; 2 Institute of Health Services Research and Health Economics, Centre for Health and Society, Medical Faculty, Heinrich Heine University Düsseldorf, Düsseldorf, Germany; 3 Institute of Family Medicine/General Practice, Medical Faculty, University of Duisburg-Essen, Essen, Germany; 4 Department of Anaesthesiology, Medical Faculty, Heinrich Heine University Düsseldorf, Düsseldorf, Germany; 5 Center of Integrated Oncology Aachen, Bonn, Cologne (CIO ABCD), Heinrich-Heine-University, Düsseldorf, Germany

**Keywords:** Trigger factors, Palliative care, Intensive care, Intensive care nurses, Interprofessional care, Interdisciplinary care

## Abstract

**Purpose:**

Triggers have been developed internationally to identify intensive care patients with palliative care needs. Due to their work, nurses are close to the patient and their perspective should therefore be included. In this study, potential triggers were first identified and then a questionnaire was developed to analyse their acceptance among German intensive care nurses.

**Methods:**

For the qualitative part of this mixed methods study, focus groups were conducted with intensive care nurses from different disciplines (surgery, neurosurgery, internal medicine), which were selected by convenience. Data were analysed using the “content-structuring content analysis” according to Kuckartz. For the quantitative study part, the thus identified triggers formed the basis for questionnaire items. The questionnaire was tested for comprehensibility in cognitive pretests and for feasibility in a pilot survey.

**Results:**

In the qualitative part six focus groups were conducted at four university hospitals. From the data four main categories (prognosis, interprofessional cooperation, relatives, patients) with three to 15 subcategories each could be identified. The nurses described situations requiring palliative care consults that related to the severity of the disease, the therapeutic course, communication within the team and between team and patient/relatives, and typical characteristics of patients and relatives. In addition, a professional conflict between nurses and physicians emerged. The questionnaire, which was developed after six cognitive interviews, consists of 32 items plus one open question. The pilot had a response rate of 76.7% (23/30), whereby 30 triggers were accepted with an agreement of ≥ 50%.

**Conclusion:**

Intensive care nurses see various triggers, with interprofessional collaboration and the patient's prognosis playing a major role. The questionnaire can be used for further surveys, e.g. interprofessional triggers could be developed.

**Supplementary Information:**

The online version contains supplementary material available at 10.1186/s13054-024-04969-1.

## Background

Intensive care units (ICU) are places of surviving in which patients in crisis situations are stabilised to such extent that discharge can be aimed for and the possibility of recovery exists [1–3]. It is often claimed that a reduction in quality of life is accepted in return. However, attempts are made to include aspects of quality of life in therapy goals and treatment planning. Patients’ values are also enquired about and should be taken into account [4].

Despite the efforts of intensive care teams, critically ill people who can no longer be cured lie in intensive care units. In addition 20% of patients die in an ICU or shortly after admission to an ICU [1, 2]. ICU patients and their relatives can therefore suffer from distressing symptoms [1, 2]. Therefore palliative care needs may arise [2]. Appropriate pain and symptom management, as well as consistent communication and decision-making, can be a challenge in patients with life-threatening illness [5]. Recommendations support the early integration of palliative care structures within the ICU setting [6–8]. Integration of such structures is possible at various levels. Primary palliative care can be provided by the ICU teams themselves without specialised palliative care (sPC) teams by addressing all palliative needs of patients and relatives. Another option would be the consultative model, in which the specialised palliative care team addresses all palliative care needs. However, the mixed model is the most recommended. Here, ICU and sPC teams work together to fulfil the palliative needs of patients and relatives [9].

Palliative care teams focus on symptom relief, effective communication about treatment goals, alignment of treatment with patients’ preferences, family support and care planning [5]. Co-treatment by multiprofessional sPC teams therefore is an appropriate option for ICU patients and can also be offered in conjunction with life-prolonging treatments and especially in collaboration with ICU teams [10–12]. Timely integration of palliative care can complement counselling, treatment and support for relatives and patients, which can lead to improved quality of life and satisfaction for both [13]. The length of stay in ICU may be reduced and inappropriate therapies avoided [2, 5]. Communication between physicians and nurses can improve and lead to higher patient and care-taker satisfaction [13].

Despite the advantages of collaboration between intensive and palliative care, difficulties of identifying patients who benefit from such shared care persist. Various studies have investigated different incentives that should lead to involvement of palliative care, including treatment preferences and options, length of stay and conflicts [5, 14–17]. Such so-called triggers were either determined in expert panels or retrospectively collected using patient data.

In Germany, these triggers were supplemented in an expert panel and checked for acceptance among ICU physicians [18]. Table 1 describes examples of possible clinical situations to which potential triggers may apply and how patients can benefit from collaboration between ICU and sPC teams.

**Table 1:** Examples for clinical situations with possible triggers and benefits

Clinical situation	Possible trigger	Possible benefit from specialized palliative care (sPC)
Relatives are informed about a possible limitation of the patient's lifetime. They perceive that the ICU team is putting a lot of effort into symptom control. Nevertheless, they would like an integration of sPC (e.g. due to own burden)	Relatives’ sPC request	The sPC team can support the ICU team in caring for relatives. Together they can provide the best possible care for those affected. Thus relatives experience support and relief
The patient suffers from complex symptoms	High symptom burden	The sPC team can use its expertise and perspective to assess symptom burden and to recommend strategies to alleviate it. Together with the ICU team and their expertise, the best possible symptom control can be achieved
The patient has an inoperable, advanced tumor. He receives maximum intensive care therapy over a longer period of time, which is continued and not adapted to any new therapy goals that may need to be achieved	Unresectable malignancy	In the case of a serious life-limiting underlying disease, sPC can be consulted regardless of the indication for intensive care. The ICU and sPC team can work together to assess the situation and realistic therapy goals. Consequently, they plan and implement treatment and care for the patient's comfort

As nurses play a major role in the care of ICU patients, they should be involved in decision-making [10] as well as in the development of triggers [15].

The aim of this study was to identify triggers that, from the perspective of German ICU nurses, should prompt the involvement of sPC for an ICU patient, and to evaluate the acceptance of these triggers.

## Methods

**Fig. 1:**
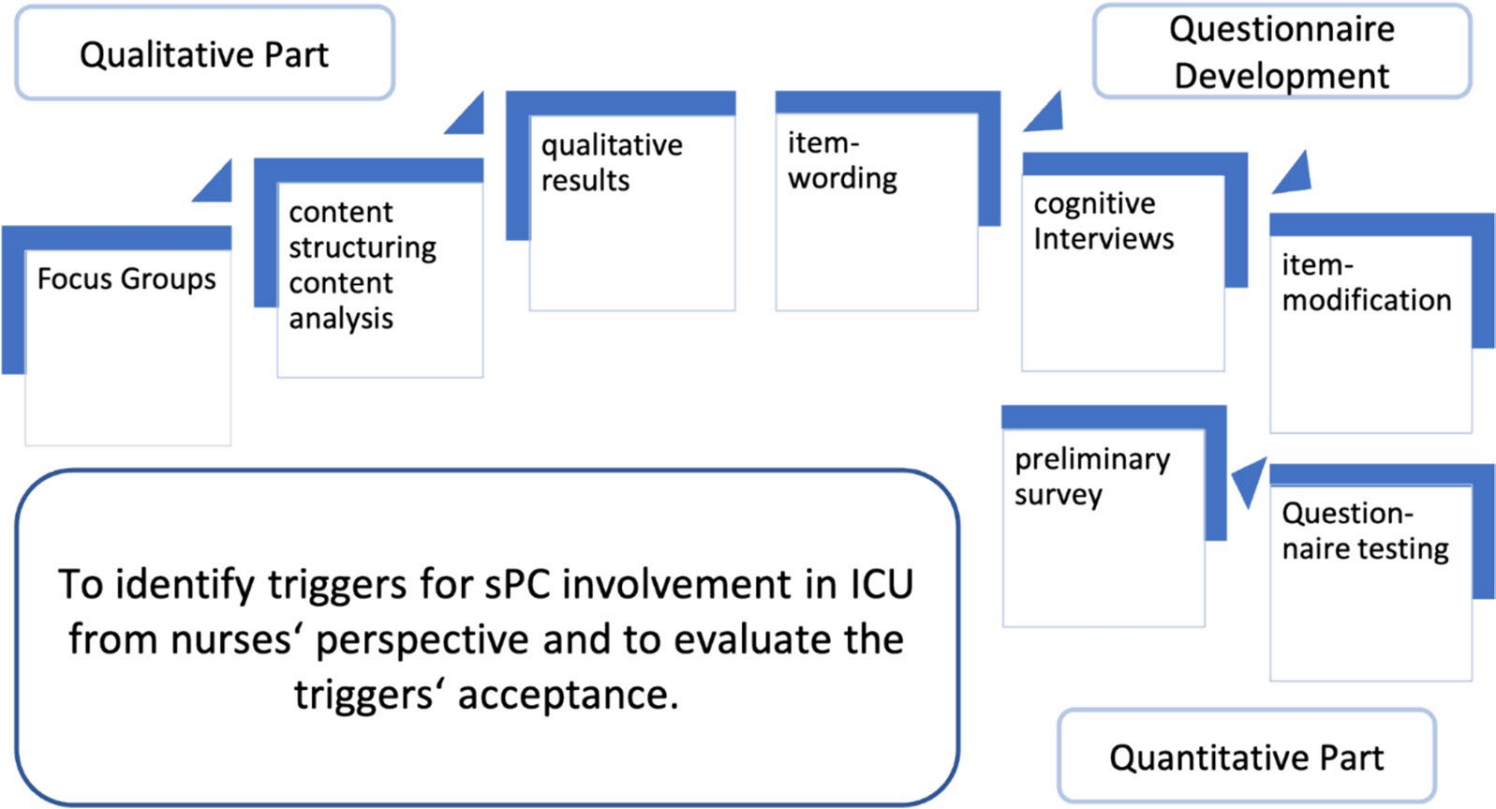
Conceptional model and study aims. *sPC* specialised palliative care, *ICU* intensive care unit

In order to achieve the study objective, focus groups were conducted to identify triggers. These were then formulated into items for a questionnaire. The questionnaire was tested and an initial analysis of the acceptance of the triggers was carried out. Figure 1 illustrates the detailed study design and objectives.

### Focus groups

Semi-structured focus groups were conducted with ICU nurses to obtain their perspectives. Potential participants were informed about the study by gatekeepers, in this case management staff, and asked to participate. Volunteers participated in the sense of a convenience sample. Nurses with at least 1 year of professional experience and current work in ICU who had contact with sPC were included. The interview guide was developed by the author team based on the research question about possible triggers for sPC involvement, including sub-questions and targeted follow-up questions. The focus groups were moderated by MS, a nurse and research assistant (Dr. PH) with experience in qualitative research and palliative and neonatal intensive care. This experience and her interest in the study—to deeper understand and strengthen the nurses' perspective—was disclosed to the participants in advance. Focus groups were recorded as audio files and transcribed according to “simple rules” [19]. No field notes were made.

Data were analysed in seven steps (initiating textwork–main categories–coding–compile passages–subcodes–coding–analysis) using the “content-structuring content analysis” according to Kuckartz [20]. Data coding was primarily done by MS using MAXQDA (Version 12, Verbi GmbH). The authors MN and JidS participated in the analysis and coding. This took place in close consultation.

### Questionnaire development

The qualitative analysis took place independently of the following step of item formulation. Nevertheless, it was the basis for developing a questionnaire to assess the acceptance of the identified triggers. These were worded into items considering the ten principles of question framing [21]. An intention was to correspond to as closely as possible to the data material. Not only the main categories and subcategories were relevant, but also the data material in the sense of the transcripts of the focus groups in order to include the content mentioned. The formulated items were repeatedly discussed in a multi-stage process within the research group with regard to appropriateness and understanding. Participants were asked for agreement on a five-point Likert scale.

In order to optimise comprehensibility, cognitive interviews were conducted to learn more about the cognitive processes that take place when answering the questions. Of interests here is how the interviewees interpret and understand terms or questions, how they recall information and events from mind, how they make decisions about how to answer and how they allocate their answers to the formal answer categories. In the present study, the techniques of inquiring for understanding, paraphrasing and thinking aloud were used [22]. When inquiring about understanding, participants are asked to describe their understanding of certain terms or the entire item. Paraphrasing asks participants to repeat an item in their own words without remembering the literal text. Thinking aloud is intended to capture the mental processes that take place when answering the items. To do this, the item is read out loud and the participants are asked to speak out their thoughts on answering. The interviews were recorded as audio files and then also transcribed according to “simple rules” [19]. When MS conducted the interviews, care was taken not to use any non-verbal amplifiers.

In this way, almost all items were assessed for understanding. Only items that seemed evident were left out, such as the question whether the patients’ age could be a trigger for involving sPC. Test persons were nurses with intensive and palliative care experience.

### Questionnaire testing with preliminary survey

Following the cognitive interview screening, a pilot was conducted in which the questionnaire was given to a small sample (n = 30) of the envisaged target group. For recruitment, head staff from three intensive care units were approached, who distributed the questionnaires to potential participants. The sample was asked to complete the questionnaire, and invited to critically comment on each item as well as at the end of the questionnaire. In addition, the time needed to complete the questionnaire was measured.

The analysis of the pilot also indicated a first tendency of the acceptance of triggers. For this purpose, as in a former study on the acceptance of triggers for sPC involvement among ICU physicians, all triggers that received at least 50% agreement were defined as accepted [18].

### Ethics

The study was approved by the Ethic Committee of the Medical Faculty of the Heinrich-Heine-University Duesseldorf, Germany (Study ID: 6114R) and conform to the Declaration of Helsinki. Written informed consent was obtained from all participants, who were all at least 18 years old.

## Results

### Focus groups

Between February 2018 and July 2019, six focus groups were conducted at four German university hospitals, involving a total of 28 ICU nurses. They represented the fields of surgery, neurosurgery and internal medicine. Three participants cancelled their participation due to illness. The duration of the focus groups, which took place in separate rooms in the work environment, was 45–90 min.

After four focus groups, there seemed to be a saturation, which became more apparent after the fifth. To strengthen this assumption, a sixth focus group took place which yielded no relevant additional information.

Since all the triggers mentioned were to be included in the evaluation and thus in the development of the questionnaire, no member check, in which the analysis is returned to the participants for review, took place.

All participants were able to report experiences of cooperation with sPC. They expressed seeing the patients’ benefit from the support, but they would like the teams to be involved earlier and more regularly. The most important element is that sPC do something good for the patients and that nurses themselves can benefit from the expertise.

Just do something good for the person for an hour (FG 1)

Simply use the expertise they bring with them (FG 6)

Overall, four main categories “prognosis, interprofessional cooperation, relatives, patients” could be formed out of the data with three to 15 subcategories each. Table 2 shows the main categories of the focus groups with examples of the related quotes and examples of the answers to the open question on piloting the questionnaire.

**Table 2:** Quotes from the main focus group categories and the open question in the pilot questionnaire

Main categories focus groups	Quote examples
Prognosis	“where it is no longer ethically tenable” (FG 3)
	“But he cannot die yet, because we simply interfere with our devices. What do we refrain from?” (FG4)
	“so above all […] the AML patients, ALL patients” (FG3)
	“for each long-term patient […] a weekly case discussion, preferably after the first week” (FG1)
Interprofessional cooperation	“Many cases we consider already a palliative care case, but the physicians do not. They continue to deliver life-sustaining treatment until the overall condition of the patients deteriorates so drastically that nothing more can be done for them, and only then they die” (FG 6)
	“There is no open discussion with the relatives. When you talk to them, they don't realise that this is the condition in which the patient will stay.” (FG3)
Relatives	“Very serious complex illness situations where the relatives have to be supported […] where someone should also be there for the relatives.” (FG1)
	“The medical treatment is exhausted, but the relatives see it differently […] Situation where relatives dictate the treatment and physicians allow it” (FG4)
Patients	“How do you take away the shortness of breath, mental problems […] if you then also ask for a specialised palliative care consultation, the psychologist will also come.” (FG 6)
	“actually mainly for very young patients”(FG 3)
Open question questionnaire	“THIS absolutely has to happen and the specialised palliative care team could mediate” (on the item Involving relatives in discussions about the further procedure)
	“Sometimes very good discussions are held, sometimes encouragement is still given where it is hopeless”

#### Prognosis

In this category, factors are named that have an influence on or are associated with a certain course and thus the prognosis. Consequently, it is about the ethical assessment of the patient's situation and the treatments undertaken, in which the ICU nurses ask for support from sPC.

SPC can support in raising comprehensive awareness of the patient's situation and enable a natural passing in the highly technical field by looking at the overall situation.

Severe pre-existing conditions such as certain oncological diseases, complications, the need for resuscitation or ventilation and length of stay in the ICU can have an influence on the prognosis and were therefore seen as triggers for the involvement of sPC.

#### Interprofessional cooperation

The participants described a potential for conflicts between ICU nurses and physicians at various points of care when nurses would like to have support from sPC.

ICU nurses prefer to be more involved in decisions, especially in order *not* to maintain life-sustaining treatment unnecessarily. In addition, they see different levels of knowledge and understanding about the work and tasks of sPC which can intensify a conflict.

Conflicts can also arise in terms of communication. The participants see that ICU physicians do not always communicate openly with relatives as well as with themselves, the nurses. This can make decision-making more difficult and increase burdens.

#### Relatives

Factors that can trigger sPC consultation can also be found in relatives. For example, they may be so burdened by the situation that they need additional support. This is particularly relevant in decision-making, where relatives may feel a sense of responsibility. SPC can also provide support in involving relatives in care processes.

Relatives` wish for palliative care counts also as a trigger for the participants. Conversely, a relative`s forceful request for ongoing life-sustaining treatment in a clear palliative situation also represents a trigger.

#### Patients

Personal characteristics of the patients can also be reasons for involving sPC.

Concerning age, sPC is more likely to be involved in the case of young patients.

### Questionnaire development

The triggers developed from the qualitative results led to 32 questionnaire items. These related to the individual patients` situation, the disease or the overall situation between the patient and the treatment team. The items were supplemented by an open free-text field in which further, unnamed triggers could be specified, as well as nine questions on demographics.

#### Cognitive interviews

In the cognitive interviews, 6 items were not tested because they were clearly understandable, such as the question about the age of the patient or the length of stay in the ICU.

In order to test the 26 other items, five cognitive interviews were conducted with one participant each, all of whom had experience in both intensive and palliative care. None of them had been part of the previous focus groups. They were asked about five or six items each in 60–90 min interviews.

The evaluation showed that four items should be modified. For example, the term “natural death” had to be defined more precisely. The remaining 22 items were understood as they were meant. The items’ interpretation by the participants was consistent to what the focus groups had described.

### Questionnaire pilot with preliminary survey

The questionnaire used for the pilot consisted of 32 triggers to be tested, one open question for triggers not mentioned, nine questions on demographics and three additional questions at the end that can give statements on the piloting of the questionnaire, i.e., a total of 45 questions.

Possible participation was openly addressed in the participating ICU teams, as was the voluntariness of participation. Due to the SARS-CoV-2 pandemic and the associated burdens as well as a general shortage of staff in nursing teams, the response rate was 76.7% of the 30 questionnaires distributed. The study team consulted with each other and decided that the number of questionnaires was sufficient for an initial survey and thus for the tendency of the results. Data on the participants are listed in Table 3.

**Table 3:** Participants in the questionnaire pilot n = 23 (in %) (*intensive care unit)

Gender	Female 14 (60.9%)	Male 9 (39.1%)	Divers 0
Department	Internal medicine 12 (52.2%)	Surgery 11 (47.8%)	
Professional experience	1–5 years 6 (26.1%)	> 5 years 17 (73.9%)	
Age	20–29 years 8 (34.8%)	30–39 years 9 (39.1%)	40–59 years 6 (26.1%)
ICU*experience	1–5 years 8 (34.8%)	> 5 years 15 (65.2%)	
Intensive care and anaesthesia training	Yes 15 (65.2%)	No 8 (34.8%)	

On average, completion of the questionnaire took 27 min. Twelve participants used the free text fields under the items (Table 2). The free comments related mainly to a reason for accepting or rejecting the particular trigger.

Some free text statements were similar to the statements in the focus groups, describing own experiences with the sPC teams.

One item which refers to the likelihood to achieve treatment goals was adjusted on the basis of the free text comments because the term “treatment goal” was perceived as ambiguous.

**Fig. 2:**
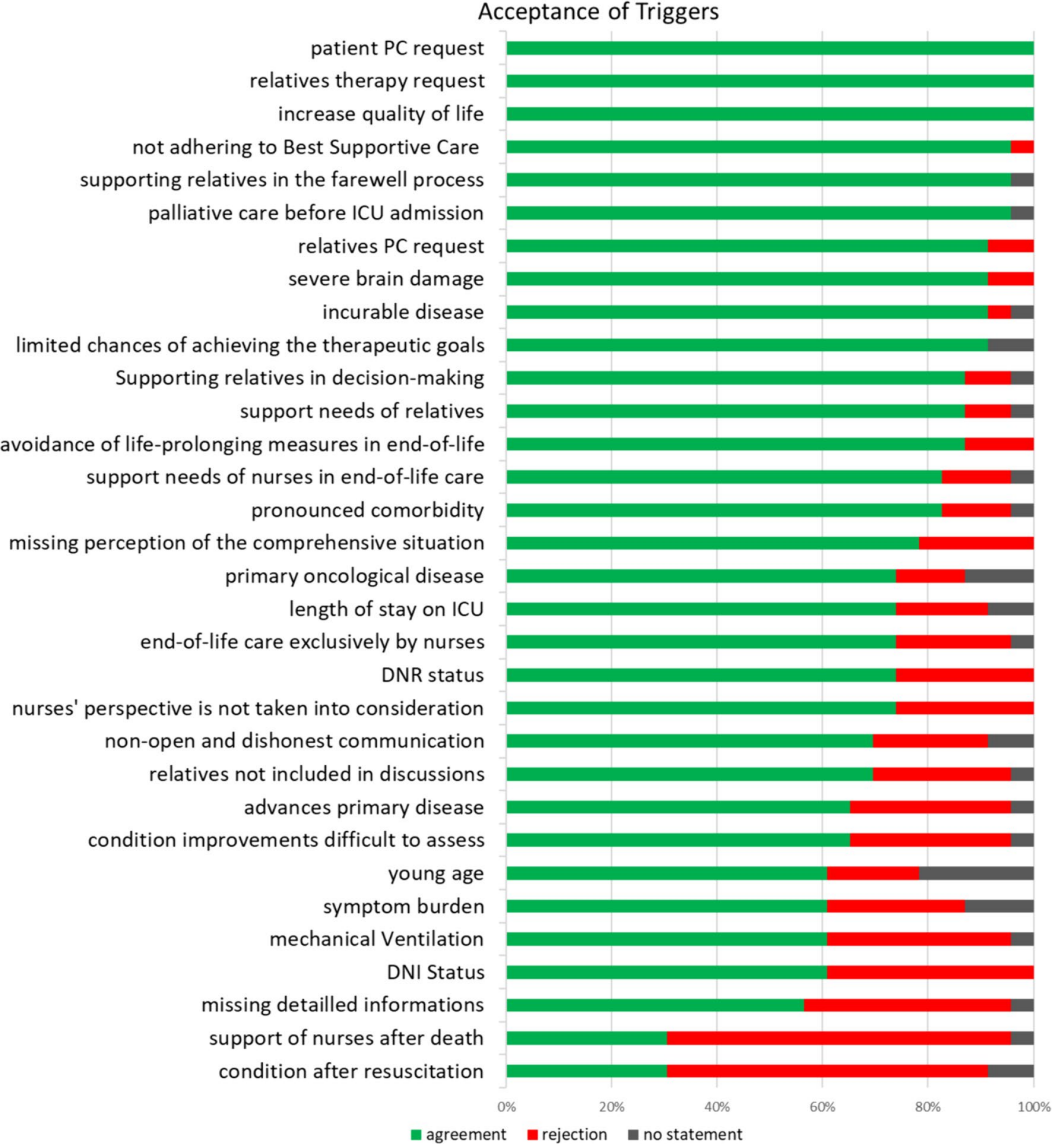
Acceptance of the triggers in the pilot. *ICU* intensive care unit, *PC* palliative care, *DNR* do not resuscitate, *DNI* no ICU transfer

The answers “fully agree” and “rather agree” were evaluated as acceptance of the respective trigger, and “rather disagree” and “strongly disagree” as rejection. “No statement” was also collected and indicated, but was not included in acceptance or rejection. With at least 50% agreement, 30 of the 32 triggers were accepted and two were rejected. Figure 2 illustrates the results of the individual items.

For further differentiation, the participants were able to provide detailed information on time and age for the questions on age, duration of ventilation and length of stay in the ICU. The median duration of ventilation was 21 days, the median duration of stay in the ICU was 14 days and the median age was < 50 years.

A further more detailed description was possible for the item symptom burden in order to name symptoms for which the participants expect a benefit from involving a sPC team. Anxiety, pain and depression were named most frequently.

## Discussion

The present study aimed to identify possible triggers for the involvement of sPC for ICU patients and their relatives from the perspective of ICU nurses. The identification of patients who benefit most from sPC involvement is important both to address the patients individual needs and to use sPC as a limited resource efficiently [9, 23]. The triggers identified in the present study relate to the individual patient situation and the support needs of patients, relatives and the intensive care nurses.

The focus groups show a conflict between physicians and nurses. Intensive care nurses feel not sufficiently involved in treatment decisions, decisions about further procedures and discussions with the patient, as mentioned in the focus groups, which leads to frustration [3]. They feel that they would involve sPC more often if they were more involved in decisions or had the opportunity to involve sPC themselves. However, physicians still want to initiate sPC involvement themselves [16].

ICU nurses ask for support from sPC in recognising and adhering to treatment limitations and in including their perceptions in decision-making processes. Especially when the prognosis is unclear, the challenge often is to recognize timely when maximum therapeutic measures represent a burdensome overtreatment that is out of proportion to the possible outcome [4]. A good ethical climate in the ICU and nursing involvement at the end of life can reduce excessive care [24]. The rejection of the trigger “support of nurses after death” is difficult to interpret. In the literature, relatives describe the situation very differently, sometimes they continue to receive support, sometimes they have the feeling that care ends quickly after death [25]. Perhaps more education is needed here about the fact that palliative care does not have to end with the death of the patient, but that it can initially accompany the relatives. ICU and sPC nurses can then provide this support together. Perhaps ICU nurses do not see any need for themselves here because they have sufficient expertise in this area and do not have any need for further support. Further consideration is required here.

Pre-existing diseases, whether related to the acute crisis or not, can have an influence on prognosis. These can include oncological diseases or brain damage like mentioned in the focus groups. Other advanced conditions such as chronic obstructive pulmonary disease or heart failure may also worsen prognosis [26, 27]. Such underlying diseases may facilitate the involvement of sPC. Here the tasks of palliative care lie in recovery and maintenance of quality of life [5, 28].

The importance of relatives in the decision to involve the sPC team is made clear in the study by the fact that they were often mentioned in the focus groups and also agreed with the items concerning them in the questionnaire. They should therefore be given prominence and taken into account not only in decision-making processes, but also in the provision of care.

They experience an exceptional situation, which can lead to increased stress and thus to an increased need for support [3]. SPC can offer additional support here, also because they can offer psycho(onco)logical support due to the multiprofessional structure. This can increase the quality of care [15].

ICU patients are often unable to communicate and thus express their wishes. As a result, relatives oftentimes communicate the patient's wishes on the basis of advance directives or presumed patient will and, if they are legal representatives, make decisions on their behalf. Therefore, they have to be adequately informed about the current condition, therapeutic options, their consequences and short- and long-term prognosis [29].

It may be helpful for them to be able to identify triggers for sPC involvement through discussion or involvement in decision-making and care. These may even be co-developed or asked for their perspective during the development. There is a need for further research.

The length of stay in an intensive care unit is mentioned as a trigger both in this study and in the literature. A stay of more than 7 days [15] or 1 month [14] is cited as a reason for sPC involvement. SPC can provide support in clarifying the prognosis, but can also be a constant reference person for relatives with frequently changing intensive care staff [30].

In the case of age, the literature tends to see old age as a trigger [5, 17]. The reason why young age is mentioned more in the focus groups may be due to the additional emotional burden felt by the caregivers.

In this study, triggers were analysed from the perspective of nurses. As nurses and physicians work together in the ICU, acceptance of the triggers by both professional groups is important.

The triggers in this study can only be partially compared with those from the literature due to the different identification. However, eight triggers—*patients’ request, relatives' request, incurable disease*, (severe) brain damage, length of stay in ICU, primary oncological disease, *(high) symptom burden*, mechanical ventilation—were tested for acceptance among German ICU physicians [18] and were also named by nursing staff in the present study. The four in italics are accepted by both professional groups and can therefore form the basis for interprofessional decisions or further studies on multiprofessional triggers.

### Limitations

It cannot be excluded that for the focus groups mainly nurses volunteered who felt that the involvement of the sPC was particularly important, and therefore had a greater interest in participating. This may mean that other perspectives are underrepresented in our sample.

Much of what is said in the focus groups can also be part of an ethics consultation [31]. Here it would be necessary to differentiate whether sPC is necessary or whether an ethic consultation can support.

The pilot was planned with 30 questionnaires in order to test the questionnaire, but also to conduct a first evaluation. Due to the tense situation, also caused by the SARS-CoV-2 pandemic, which led to enormous stress for the carers, this number was not reached. The response rate of 23 questionnaires allows no more than an explorative analysis of pure agreement versus rejection.

The participants in this study were ICU nurses. The results show that relatives are highly relevant. They and their perspective should therefore be the target group of future studies on this topic.

## Conclusion

The nurses interviewed in the focus groups see a clear benefit for those affected in the shared care provided by sPC. In the perspective of ICU nurses, the relevant functions of sPC can be both advisory and (co-)treating.

With the formulated triggers, they can now bring this into decision-making processes. A shared exchange based on the triggers can also support the conflict described.

The piloted questionnaire can now be used to test the triggers for acceptance in a larger cohort. The initial evaluation confirms the triggers brought forward in the focus groups. A cross-professional survey is also possible to develop multi-professional triggers.

### Supplementary Information

Supplementary Material 1.

Supplementary Material 2.

Supplementary Material 3.

Supplementary Material 4.

Supplementary Material 5.

## Data Availability

The datasets generated and analysed during the current study are not publicly available due the extend but are available from the corresponding author on reasonable request.

## Abbreviations

ICU: Intensive care unit
sPC: Specialised palliative care
Dr. PH: Doctoral degree in public health
