# Supplementary material for: Triggers of intensive care patients with palliative care needs from nurses’ perspective: a mixed methods study
Source: Crit Care. 2024 May 28;28:181. doi: 10.1186/s13054-024-04969-1 (PMC11134896; doi:10.1186/s13054-024-04969-1)
Supplement: Supplementary file 1 — Supplementary Material 1. [file 13054_2024_4969_MOESM1_ESM.pdf]

**N = follow-up questions on understanding**
After being asked the item question, the respondent should describe how they understand certain aspects or terms.
**P = Paraphrasing**
After being asked the item question, the interviewee should reproduce it in their own words without reproducing the text verbatim.
**th a = think aloud**
After being asked the item question, the interviewee is asked to formulate aloud all the thoughts that lead him/her to answer the question. The interviewer should point out the need to think aloud during longer pauses.
This technique should be practised in advance with the interviewee using an example. Example practice question:
"Please imagine your flat/house. How many windows are there? Please tell me what you see and think as you count the windows."
"How often have you been to the dentist in the last 12 months?"
"Please think about the days you watch TV. How long in hours and minutes do you watch TV in a day?"

In the following, I will read out items that describe possible situations for patients in intensive care units. The situations are to be rated from strongly agree to strongly disagree as to whether the involvement of a palliative care team is recommended. After reading out the item, I will ask you questions relating to the response to the item.

<u>Participant 1:</u>
**1) Item 1 P + N**
The prospect that the therapy goal can still be achieved for a patient is too low.
Questions:
- Please describe the situation in your own words.
- How do you understand the word "therapy goal"?
- What do you mean when you say that a treatment goal can no longer be achieved?

**2) Item 2 th a**
The patient is no longer perceived as a whole (complications, other illnesses, social environment, etc.), but is focussed on the intensive care indication.
Prompt:
- Please state aloud any thoughts you have about assessing the situation.

**3) Item 3 P + N**
The palliative care team can provide support to enable a patient to die naturally in the final phase.
Questions:
- Please describe the situation in your own words.
- What does "terminal phase" mean to you?
- What does "natural death" mean to you?

**4) Item 4 P + N**
It is difficult to assess whether the patient's condition can improve.
Questions:
- Please describe the situation in your own words.
- What do you understand by "improve" for patients in intensive care?

**5) Item 5 th a**
A concept of Best Supportive Care that has already been decided upon is not consistently adhered to, i.e. therapies are continued or restarted.
Prompt:
- Please state aloud any thoughts you have when assessing the situation.

<u>Participant 2</u>
**6) Item 6 th a**
You feel that communication with patients and relatives is not open and honest enough.
Prompt:
- Please state aloud any thoughts you have about assessing the situation.

**7) Item 7 th a**
In your opinion, relatives and patients are not given enough detailed information about the illness and prognosis.
Prompt:
- Please state aloud any thoughts you have about assessing the situation.

**8) Item 8 P + N**
The carer's assessment of a patient situation is not taken into account in discussions about decisions and treatment procedures.
Questions:
- Please describe the situation in your own words.
- How do you understand that the carer's assessment is not taken into account?
- What could discussions about decisions include for you?
- What could include discussions about treatment procedures for you?

**9) Item 9 th a**
The care team has its own need for support or guidance in the end-of-life care of patients.
Prompt:
- Please state aloud any thoughts you have about assessing the situation.

**10) Item 10 P + N**
The nursing team needs support or guidance in the care of patients after death.
Questions:
- Please describe the situation in your own words.
- What do you understand by need for support?
- What do you understand by the need for guidance?
- What do you understand by care after death?

Participant 3

**11) Item 11 th a**

The team agrees that the patient's underlying disease is at an advanced stage.

Prompt:

- Please state aloud any thoughts you have about assessing the situation.

**12) Item 14 P + N**

The patient has severe brain damage so that neurological recovery is unlikely.

Questions:

- Please describe the situation in your own words.
- What do you understand by severe brain damage?
- What exactly does "severe" mean to you in this context?
- What do you understand by "neurological recovery"?
- How would you categorise "unlikely" in this context?

**13) Item 15 P + N**

The patient is known to have an incurable disease with a poor prognosis.

Questions:

- Please describe the situation in your own words.
- What do you understand by "incurable disease"?
- What do you understand by "poor prognosis"?

**14) Item 16 P + N**

The patient has an underlying oncological disease.

Questions:

- Please describe the situation in your own words.
- What is an underlying oncological disease for you?
- Do you have any other associations with the word "underlying disease"?

**15) Item 17 P + N**

The patient was already receiving palliative care before admission to the ICU.

Questions:

- Please describe the situation in your own words.
- How do you understand the previous palliative care?

Participant 4

**16) Item 18 P + N**

The patient has a pronounced comorbidity.

Questions:

- Please describe the situation in your own words.
- How do you understand "pronounced comorbidity"?

**17) Item 20 th a**

End-of-life care is provided exclusively by you as a carer, while you have the feeling that your medical colleagues are withdrawing.

Prompt:

- Please now state aloud any thoughts you have about assessing the situation.

**18) Item 21 P + N**
The patient's relatives need more support than can be provided in everyday life in the intensive care unit.
Questions:
- Please describe the situation in your own words.
- What do you understand by "more support than can be given in everyday life in the intensive care unit"?
- What do you associate with "support" in relation to relatives of patients in the ICU?

**19) Item 22 th a**
Relatives are not involved in discussions about how to proceed with the patient.
Prompt:
- Please now reproduce aloud all the considerations you have to assess the situation.

**20) Item 23 P + N**
Relatives need support to be able to make meaningful decisions for the patient.
Questions:
-Please describe the situation described in your own words.
- What do you mean by "decisions with consequences for the patient"?
- What do you associate with support for relatives in this context?

<u>Participant 5</u>
**21) Item 24 P + N**
Contrary to medical indications, the relatives have a high desire for therapy.
Questions:
- Please describe the situation described in your own words.
- How do you understand "against medical indications"?
- What do you mean by a high desire for therapy?

**22) Item 26 P + N**
Relatives need support during parting processes if the patient is about to die.
Questions:
- Please describe the situation described in your own words.
- What do you mean by "death for the foreseeable future"?
- What do "farewell processes" mean to you?
- What do you mean by "support" in this context?

**23) Item 29 P + N**
**The patient has a DNR (do not resuscitate) status.**
Questions:
- Please describe the situation described in your own words.
- What do you mean by DNR status?

**24) Item 30 P + N**
The patient has a DNI (do not intubate) status.
Questions:
- Please describe the situation described in your own words.
- What do you mean by DNI status?

**25) Item 31 th a**
The quality of life of a patient is particularly impaired and should be increased by small measures such as massages, transfer to the outside, being, reading etc.
Prompt:
- Please now reproduce aloud all the considerations you have to assess the situation.

**26) Item 32 P + N**
The patient suffers from severe physical and/or psychological symptoms.
Questions:
- Please describe the situation described in your own words.
- What do you mean by “heavy burden of symptoms”?
- What do you mean by a physical burden of symptoms?
- What do you mean by psychological burden of symptoms?
