# Supplementary material for: Triggers of intensive care patients with palliative care needs from nurses’ perspective: a mixed methods study
Source: Crit Care. 2024 May 28;28:181. doi: 10.1186/s13054-024-04969-1 (PMC11134896; doi:10.1186/s13054-024-04969-1)
Supplement: Supplementary file 2 — Supplementary Material 2. [file 13054_2024_4969_MOESM2_ESM.pdf]

1. are there palliative consultations on your ward? yes/no

If yes, how often do they occur? ___ times per month / or per year

How many palliative consultations do you think you have seen in the last 6 months?

2. if you think about the palliative consultations you have seen in the last 12 months: What kind of patients were they, and what were the reasons or triggers for making a palliative care consult for these patients? Please tell us!

3. thank you. I understand that you have observed the following triggers for palliative consultations: ... (X-Y-Z). Are there any other triggers for palliative consultations...?

4. if you think about the patients you have just described: How do you feel about palliative consultations being requested for these patients? Should the reasons or triggers you described also lead to a palliative consultation being requested in future? Why?

5. can you describe situations in which, in your opinion, patients would have benefited from a palliative care consultation but it was not requested?

6. why do you think these patients would have benefited from a palliative care consultation?

7. if you were allowed to decide on your own: When or for which patients would you provide a palliative care consult?

8. are there any other characteristics that you wish would lead to the request for a palliative care consultation?

9.would you like to tell me anything else about the triggers that could or should trigger a palliative care consultation for intensive care patients?
