# Supplementary material for: Triggers of intensive care patients with palliative care needs from nurses’ perspective: a mixed methods study
Source: Crit Care. 2024 May 28;28:181. doi: 10.1186/s13054-024-04969-1 (PMC11134896; doi:10.1186/s13054-024-04969-1)
Supplement: Supplementary file 3 — Supplementary Material 3. [file 13054_2024_4969_MOESM3_ESM.pdf]

Table 2: main and subcategories (*Intensive Care Unit)

main categories	subcategories
prognosis	ethical assessment of the patient situation<br>ethical assessment of the therapy<br>therapeutic limits<br>natural dying<br>overall awareness of the patient's situation<br>decision-making<br>consistency in the carrying out of decisions<br>progression of the disease<br>mechanical ventilation<br>brain injury<br>inauspicious prognosis<br>certain oncological diseases<br>complications<br>need for resuscitation<br>length of stay in ICU*
interprofessional cooperation	Involvement of nurses<br>Communication<br>Knowledge about palliative care
relatives	intense burden<br>inclusion in care<br>decision-making<br>Wish for life-sustaining treatment<br>Wish for palliative care
patients	age<br>wish for palliative care<br>symptom burden
