# Supplementary material for: Triggers of intensive care patients with palliative care needs from nurses’ perspective: a mixed methods study
Source: Crit Care. 2024 May 28;28:181. doi: 10.1186/s13054-024-04969-1 (PMC11134896; doi:10.1186/s13054-024-04969-1)
Supplement: Supplementary file 4 — Supplementary Material 4. [file 13054_2024_4969_MOESM4_ESM.pdf]

The palliative care consultation in the intensive care unit

The following section describes patient situations that may occur in your intensive care unit. For each statement, please indicate whether you would be in favour of involving the palliative care team in the respective case. It is irrelevant here whether it is a question of palliative care and support for the patient and their relatives or an advisory role for the palliative care team.

For reasons of better readability, the simultaneous use of the language forms male, female and diverse (m/f/d) is avoided. All personal designations apply equally to all genders

	**In the following situation, I am in favour of calling in a palliative care team.**	**fully agree**	**rather agree**	**rather disagree**	**disagree**	**no response**
1	You consider the treatment goal in terms of cure to be too high and think an adjustment is necessary.					
2	The patient is no longer perceived as a whole (complications, other illnesses, social environment, etc.), but is reduced to the indication for intensive care.					
3	The palliative care team can provide support to ensure that life-prolonging measures are neither taken nor continued for a patient in the terminal phase.					
4	It is difficult to assess whether the patient's condition can improve.					
5	An already agreed concept in terms of Best Supportive Care is not consistently adhered to, i.e. therapies are continued or restarted.					
6	You feel that communication with patients and relatives is not open and honest enough.					
7	In your opinion, relatives and patients are not given enough detailed information about the disease and prognosis.					
8	The carer's assessment of a patient situation is not taken into account in discussions about decisions and treatment procedures.					
9	The care team has its own need for support or guidance in providing care in the final phase.					
10	The care team has its own need for support or instruction in the care of patients after death.					
11	The team agrees that the patient's underlying disease is at an advanced stage.					

	**In the following situation, I am in favour of calling in a palliative care team.**	**fully agree**	**rather agree**	**rather disagree**	**disagree**	**no response**
12	The patient requires long-term ventilation.<br><br>If you have answered here with "I tend to agree" or "I agree<br>fully agree", please indicate in the following list<br>the following list what "long-term" means to you.<br>means to you.<br>☐ from 7 days<br>☐ from 14 days<br>☐ from 21 days<br>☐ ☐ from 28 days					
13	The patient has been in intensive care for x days.<br><br>If you answered "somewhat agree" or "agree<br>fully agree", please indicate from which treatment<br>treatment period would be a trigger factor for you?<br>trigger factor for you?<br>☐ 7 days<br>☐ 14 days<br>☐ 21 days<br>☐ 28 days<br>☐ 50 days<br>☐ 100 days					
14	The patient has severe brain damage, so that neurological recovery is not seen on the basis of the diagnosis.					
15	The patient has an incurable disease with a poor prognosis.					
16	The patient has an underlying oncological disease.					
17	The patient had already received palliative care before admission to the ICU.					
18	The patient has a pronounced comorbidity.					
19	The patient was resuscitated.					
20	Care in the final phase is provided exclusively by you as a carer, while you have the feeling that your medical colleagues are withdrawing.					

	**In the following situation, I am in favour of calling in a palliative care team.**	**fully agree**	**rather agree**	**rather disagree**	**disagree**	**no response**
21	The patient's relatives need more support than can be provided in everyday life in the intensive care unit.					
22	Relatives are not involved in discussions about how to proceed with the patient.					
23	Relatives need support to be able to make important decisions for the patient.					
24	Contrary to medical indications, the relatives have a strong desire for therapy.					
25	The relatives express the wish for palliative co-treatment.					
26	Relatives need support in the process of saying goodbye when the patient is likely to die.					
27	The patient still appears young to you.<br><br>If you answered "strongly agree" or "somewhat agree", please<br>agree", please indicate in the following<br>the following lists, please indicate what you consider "young"<br>describes for you.<br>☐ <30 years old<br>☐ <40 years<br>☐ <50 years<br>☐ <60 years<br>☐ <70 years<br>☐ <80 years					
28	The patient expresses the wish for palliative co-treatment or has expressed this wish in an advance directive.					
29	The patient has a DNR (do not resuscitate) status.					
30	The patient has a DNI (do not intubate) status.					
31	A patient's quality of life is particularly impaired and should be improved by small measures such as massages, transfers outside, being there, reading aloud, etc.					

	**In the following situation, I am in favour of calling in a palliative care team.**	**fully agree**	**rather agree**	**rather disagree**	**disagree**	**no response**
32	The patient is suffering from severe physical and/or psychological symptoms.<br>If you have answered yes, please indicate in the following field which symptoms this could be.					

33. Are there any other situations that have not been described above, but which you would describe as a reason for calling in a palliative care team? Please enter these situations in the following free text field!

Please tick the appropriate boxes in the following questions about yourself.

34 How old are you?
o < 20 years
o 20 - 29 years old
o 30 - 39 years
o 40 - 49 years
o 50 - 59 years
o > 60 years

35 Which gender are you?
o Female
o Male
o Diverse

36. which denomination do you belong to?
o Roman Catholic
o Protestant
o Orthodox
o Jewish
o Muslim
o Buddhist
o Other denomination, please specify ________________________________
o No denomination

37 Are you a practising believer?
o Yes
o No

38. how long have you been working in the care sector? (without training period)
o < 1 year
o 1 - 5 years
o > 5 years

39. have you completed any of the following further or advanced training courses? If yes, please indicate how much time has passed since you completed it.
Specialist nurse for anaesthesia and intensive care
o Yes
□ < 1 year
□ 1- 5 years
□ > 5 years
o No

Nursing care in oncology
o Yes
□ < 1 year
□ 1- 5 years
□ > 5 years
o No

Palliative care course for carers
o Yes
□ < 1 year
□ 1- 5 years

□ > 5 years
o No

Please specify other: ___________________________

40 How long have you been working in intensive care?
o < 1 year
o 1 - 5 years
o > 5 years

41 Which specialist department does the intensive care unit you are currently working in belong to?
o Internal medicine, more detailed information on the speciality if required:
____
o Surgery, more detailed information on the speciality if required:
____
o Interdisciplinary
o Anaesthesiology

42 Which level of care does the hospital where you work belong to?
o University hospital
o Other maximum care hospital
o Basic and standard care hospital

This concludes your participation in the questionnaire.
Thank you very much for your participation!
