# Supplementary material for: Triggers of intensive care patients with palliative care needs from nurses’ perspective: a mixed methods study
Source: Crit Care. 2024 May 28;28:181. doi: 10.1186/s13054-024-04969-1 (PMC11134896; doi:10.1186/s13054-024-04969-1)
Supplement: Supplementary file 5 — Supplementary Material 5. [file 13054_2024_4969_MOESM5_ESM.pdf]

Composition of the focus groups

focus groups at four university hospitals	total participants (of whom with further training in intensive care)	Departments
1	9 (5)	surgery
2	4 (3)	neurosurgery and surgery
3	5 (3)	internal medicine
4	2 (2)	internal medicine
5	3 (2)	neurosurgery
6	5 (2)	internal medicine
